# Whole-Body ARHGAP21-Deficiency Improves Energetic Homeostasis in Lean and Obese Mice

**DOI:** 10.3389/fendo.2019.00338

**Published:** 2019-05-29

**Authors:** Gabriela Moreira Soares, Lucas Zangerolamo, Jose Maria Costa-Júnior, Jean Franciesco Vettorazzi, Everardo Magalhães Carneiro, Sara Teresinha Saad, Antonio Carlos Boschero, Helena Cristina Barbosa-Sampaio

**Affiliations:** ^1^Obesity and Comorbidities Research Center, Institute of Biology, University of Campinas/UNICAMP, Campinas, Brazil; ^2^Department of Structural and Functional Biology, Institute of Biology, University of Campinas/UNICAMP, Campinas, Brazil; ^3^Hematology and Hemotherapy Center, University of Campinas, HEMOCENTRO-UNICAMP, Campinas, Brazil

**Keywords:** ARHGAP21, Rho-GAP, energy homeostasis, food intake, obesity

## Abstract

Inhibition of Rab-GAP TBC1 domain family member 1 (TBC1D1) reduces body weight and increases energy expenditure in mice. Here, we assessed the possible involvement of GTPase activating protein 21 (ARHGAP21), a Rho-GAP protein, in energy homeostasis. Wild-type and whole-body ARHGAP21-haplodeficient mice were fed either chow or high-fat diet for 10 weeks. These mice were analyzed for body weight, food intake, voluntary physical activity, and energy expenditure by indirect calorimetry. Real-time PCR was performed to determine changes in the expression of hypothalamic-anorexic genes. Whole-body ARHGAP21-haplodeficient mice showed lower body weight and food intake associated with increased energy expenditure. These mice also showed higher expression of hypothalamic-anorexic genes such as POMC and CART. Our data suggest that the reduction in body weight of ARHGAP21-haplodeficient mice was related to alterations in the central nervous system. This suggests a new role for ARHGAP21 in energetic metabolism and prompts us to consider GAP protein members as possible targets for the prevention and treatment of obesity and related diseases.

## Introduction

Energy homeostasis depends on a balance between food intake and energy expenditure regulated by complex physiological mechanisms. A disturbance in these processes can lead to obesity (1, 2). Obesity and overweight are pandemic, affecting more than 2 billion people worldwide (3, 4). The hypothalamus plays an important role in this context, controlling feeding behavior, and energy metabolism through a complex network of neurons that express distinct neurotransmitters (5, 6).

Insulin and leptin signaling, as well as the POMC-NPY axis, are among the canonical molecular pathways that control energy intake and expenditure (7, 8). Insulin, just before a meal, inhibits food intake by activating anorexigenic genes in hypothalamus. On the other hand, leptin acts regulating food intake and energy expenditure, this hormone is secreted by the adipose tissue, in order to estimate body energetic pads (9).

Actually, many proteins have been proposed to regulate food intake and energetic expenditure, and other less-studied molecules also appear to be involved (10). Of these, the GTPase activating proteins (GAPs) emerge as possible modulators of energy homeostasis. GAP proteins regulate the activity of small G proteins, in general, accelerating their return to inactive state, through the induction of GTP hydrolysis (11). According to protein subdomains, small G proteins are classified into five families: Ras, Rho, Rab, Arf, and Ran (12), all of them are mainly involved in cytoskeletal rearrangement and trafficking of vesicles to the membrane in various cell types (13, 14). Each small G protein has its own GAP, which regulates the activity and function of the GTPase. In central nervous system, Rho GTPases regulate neuronal migration and growth, as well as synaptic transmission (15), and recently, some GAP proteins have been explored in the metabolic context, demonstrating an important role in glycemic and energetic homeostasis.

Indeed, GAP TBC1 domain family member 1 (TBC1D1)-deficiency reduces body weight (16–20), decreases respiratory quotient (16–19) and increases energy expenditure (17–20) in addition to suppressing diet-induced obesity (16, 19). Recently, our group reported that reduction of ARHGAP21 (a Rho-GAP isoform) improved glucose tolerance and insulin sensitivity and reduced weight gain in mice fed on high-fat diet (21). However, the role of ARHGAP21 in hypothalamic appetite control and whole-body energy homeostasis remains unclear.

Here, we observed that whole-body ARHGAP21-deficiency reduced fat pad depots as well as body weight, probably increasing the expression of the anorexic genes POMC and CART in the hypothalamus, genes associated with reduction in food consumption and increments of energy expenditure. These findings explain, at least in part, why Het-HFD mice did not become obese, highlighting GAP protein members as important targets for the prevention and control of obesity and associated diseases.

## Materials and Methods

### Animals

The haplodeficient mouse (Het) is a whole-body ARHGAP21 gene-deficiency model, expressing ~50% ARHGAP21. The generation and genotyping of ARHGAP21-haplodeficient mice were performed as previously described (22). Paired male wild-type littermates were used as controls (Ctl). All mice were maintained at 22 ± 1°C on a 12-h light–dark cycle with free access to food and water. At 1 month of age, the mice received chow (Ctl and Het) or a high-fat diet, (Ctl-HFD and Het-HFD). This diet composition was described previously (23). Mice from all groups were allowed to feed and drink tap water for 10 weeks *ad libitum*. All experiments involving animals were approved by the Animal Care Committee at UNICAMP (approval number: 3783-1).

### Body Parameters

The body weights of all mice were evaluated once a week during the 10 weeks of diet treatments (*n* = 6). In addition, the perigonadal fat pad and the interscapular brown adipose tissue (BAT) were dissected and weighed (*n* = 6). BAT and hypothalamus samples were separated for RNA extraction.

### Food Intake

At the 9th week of treatment, mice were maintained, individually, in home cages for 24 h of adaptation (*n* = 3–5). After that, food consumption was measured during 3 consecutive days and was calculated by the difference between the food weight at 7 p. m. vs. 7 a. m. Food intake was then determined as the mean food consumption of this period (24, 25).

### Indirect Calorimetry

Metabolic rates were measured by indirect calorimetry using an open-circuit indirect calorimeter system, the Comprehensive Lab Animal Monitoring System: Oxymax-CLAMS (Columbus Instruments, Columbus, OH, USA). At the 10th week of treatment, mice were acclimated for 24 h in the system cages (*n* = 3), and the Oxymax-CLAMS was calibrated as recommended by the manufacturer. After the acclimation period, the rate of oxygen consumption (VO_2_), respiratory exchange ratio (RER), heat rate (Kcal/h), and ambulatory activity (measured as total beam breaks which means, the sum of x, y, and z axis) were measured during the light and dark periods (26). These data were acquired for 24 h and were analyzed using Oxymax Windows software (Columbus Instruments, Columbus, OH, USA).

### Serum Leptin Measurement

The serum samples were obtained by centrifugation of blood samples (1,100 g for 15 min at 4°C) and were stored at −80°C for posterior leptin quantification. Leptin concentration was measured using Mouse Leptin ELISA Kit (Cat. EZML-82K, Merck Millipore, Darmstadt, Germany), according to the manufacturer's instructions (*n* = 5–6).

### mRNA Isolation and Real Time Quantitative PCR

The total RNA content of the perigonadal adipose tissue (*n* = 5–6), BAT (*n* = 3–4), and hypothalamus (*n* = 5–6) was extracted using TRIzol reagent (Life Technologies, Gaithersburg, MD, USA), following phenol-chloroform RNA extraction, according to the manufacturer's recommendations. RNA concentration was measured by Nanodrop (Nanodrop Thermo scientific, Wilmington, DE, USA). cDNA was prepared using 1 μg RNA and MultiScribe reverse transcriptase (Applied Biosystems, Foster City, CA, USA). The SYBR-green master mix (Applied Biosystems, Foster City, CA, USA) was used in the PCR reactions. Quantification was performed using the 7500 Fast Real-time PCR System (Applied Biosystems, Foster City, CA, USA). The specificities of amplifications were verified by melting-curve analyses. The relative expression of mRNAs was determined after normalization with GAPDH, using the 2-ΔΔCt method. Primer sequences used for real-time PCR assays were as follows: ARHGAP21 forward: 5′-tcatgcctgtgtgcataccc-3′, ARHGAP21 reverse: 5′-aagctcccaacagtgcaaac-3′; Leptin forward: 5′-gagacccctgtgtcggttc-3′; Leptin reverse: 5′-ctgcgtgtgtgaaatgtcattg-3′; POMC forward: 5′-ggcttgcaaactcgacctc-3′, POMC reverse: 5′-tgacccatgacgtacttccg-3′; CART forward: 5′-acctttgctgggtgcccgtg-3′, CART reverse: 5′-tgcaacgcttcgatcagctcc-3′; NPY forward: 5′-tactccgctctgcgacacta-3′, NPY reverse: 5′-tcttcaagccttgttctggg-3′; AgRP forward: 5′-gagttcccaggtctaagtctgaatg-3′, AgRP reverse: 5′-atctagcacctccgccaaag-3′; UCP1 forward: 5′-ctgccaggacagtacccaag-3′, UCP1 reverse: 5′-tcagctgttcaaagcacaca-3′; GAPDH forward: 5′-cctgcaccaccaactgctta-3′, GAPDH reverse: 5′-gccccacggccatcacgcca-3′.

### Western Blot Analysis

The BAT lysates (*n* = 3–4) were prepared using TissueLyser LT (Qiagen, Hilden, Germany) and then were placed in a 1.5 ml tube and mixed with a lysis/antiprotease buffer containing 7 mol/L urea, 2 mol/L thiourea, 100 mmol/L Tris pH 7.5, 10 mmol/L sodium pyrophosphate, 100 mmol/L sodium fluoride, 10 mmol/L ethylenediamine tetraacetic acid (EDTA), 10 mmol/L sodium vanadate, 2 mmol/L phenylmethylsulfonyl fluoride (PMSF), and 1% Triton X100. The extracts were then centrifuged at 12,600 g at 4°C for 40 min to remove insoluble materials. The protein concentration of the supernatants was assayed using the Bradford dye method (27), using bovine serum albumin (BSA) as a standard curve and the Bradford reagent (Bio-Agency Lab., São Paulo, Brazil). For SDS (sodium dodecyl sulfate) polyacrylamide gel electrophoresis, all samples were treated with a Laemmli buffer containing dithiothreitol. After heating to 100°C for 5 min, proteins were separated by electrophoresis in a 12% polyacrylamide gel. The transfer to nitrocellulose membranes was performed in a Trans Blot transfer for 2 h in 100 V, with a tris/glycine buffer. After, the membranes were blocked with 5% BSA for 1 h and were then incubated with specific antibodies—UCP1 (#14670; Cell Signaling Technology, Danvers, MA), GAPDH (G9545; Sigma, St. Louis, Missouri, USA)—that were diluted 1:1,000 and subsequently detected by exposure to chemiluminescent substances (luminol and peroxidase). After incubation, the appropriate secondary antibody (dilution 1:10,000; Invitrogen, São Paulo, Brazil) was added for further luminescence detection followed by detection in Amersham Imager 600 (GE Healthcare Life Sciences, Buckinghamshire, UK). The quantification of the bands was performed by densitometry using the ImageJ software (National Institutes of Health, Bethesda, MD, USA).

### Statistical Analysis

The data were analyzed by Student's *t*-test (GraphPad Prism 5, La Jolla, CA, USA) and were presented as the means ± standard errors media (SEM). The differences between groups were considered statistically significant if *P* ≤ 0.05.

## Results

### Anorexigenic Effects of Whole-Body ARHGAP21 Reduction in Het Mice

**Figure 1:**
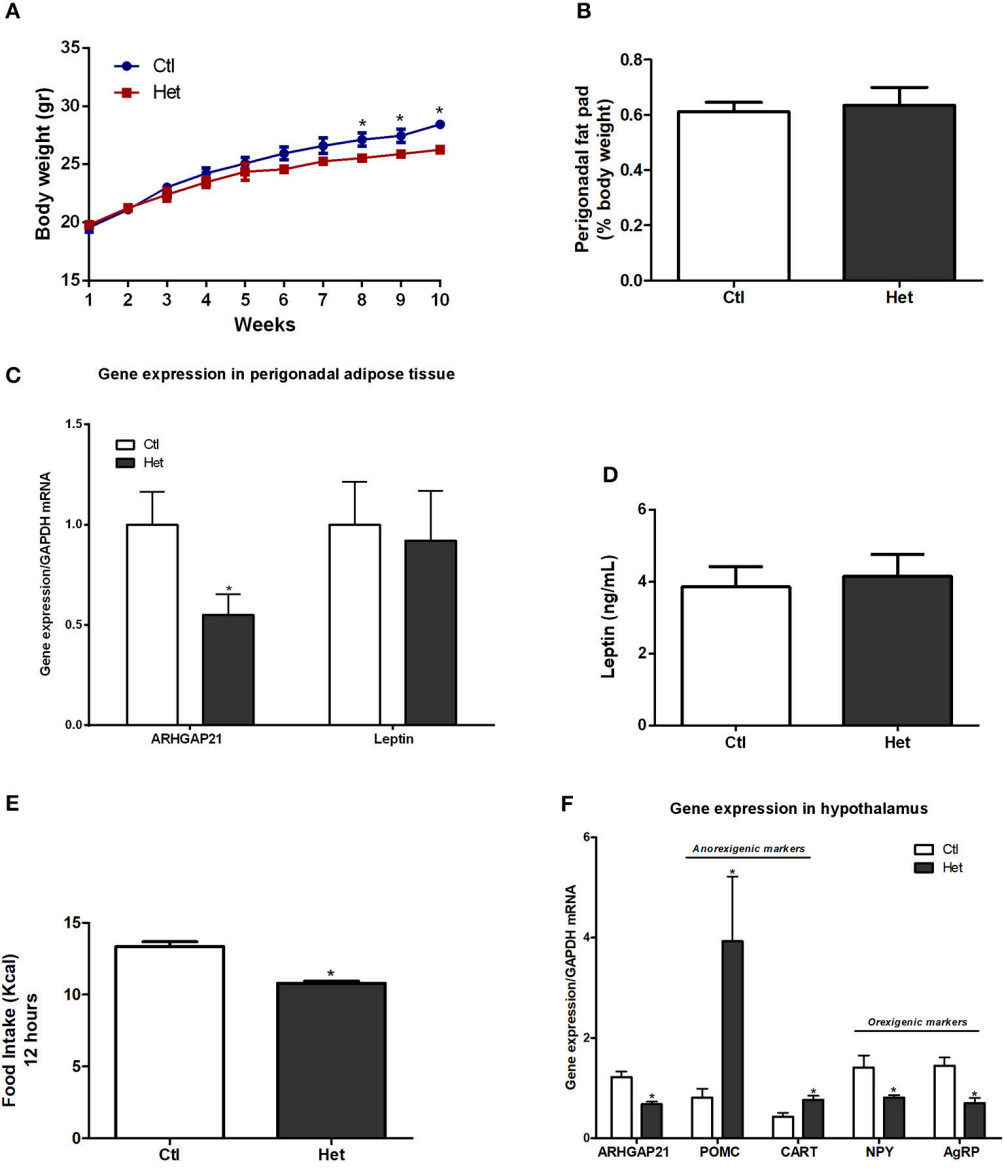
Anorexigenic effects of whole-body ARHGAP21 reduction in Het mice. Body weight curve **(A)** *n* = 6. Perigonadal fat pad weight **(B)** *n* = 6. Real-time PCR assay of ARHGAP21 and leptin mRNA levels in perigonadal adipose tissue **(C)** *n* = 5–6. Serum leptin level **(D)** *n* = 5. Food intake over 12 h **(E)** *n* = 3–4. Real time PCR assay of hypothalamic ARHGAP21, POMC, CART, NPY, and AgRP mRNA levels **(F)** *n* = 5–6. Control mice (Ctl) and ARHGAP21-haplodeficient mice (Het) fed a chow diet for 10 weeks. Data are the mean ± SEM. *P ≤ 0.05 (Student's-*t*-Test).

The body weight of mice was measured once per week for 10 weeks. At the 8th week, Het mice displayed lower body weight than did the Ctl mice until the end of the experimental period (Figure 1A). The weight of the perigonadal fat pad of Het was similar to that of Ctl mice (Figure 1B), as well as the mRNA levels of leptin in this tissue and serum leptin levels (Figures 1C,D). Also, ARHGAP21 mRNA content in the perigonadal adipose tissue of Het mice was reduced (Figure 1C). Het mice showed lower food intake than did Ctl mice (Figure 1E). Consistent with these findings, Het mice displayed significant increases in hypothalamic mRNA levels of the anorexigenic markers POMC and CART and reductions in the mRNA levels of the orexigenic markers NPY and agRP (Figure 1F). We also found that the ARHGAP21 mRNA content was lower in the hypothalamus of Het mice (Figure 1F).

### Energy Homeostasis of ARHGAP21 Het Mice

**Figure 2:**
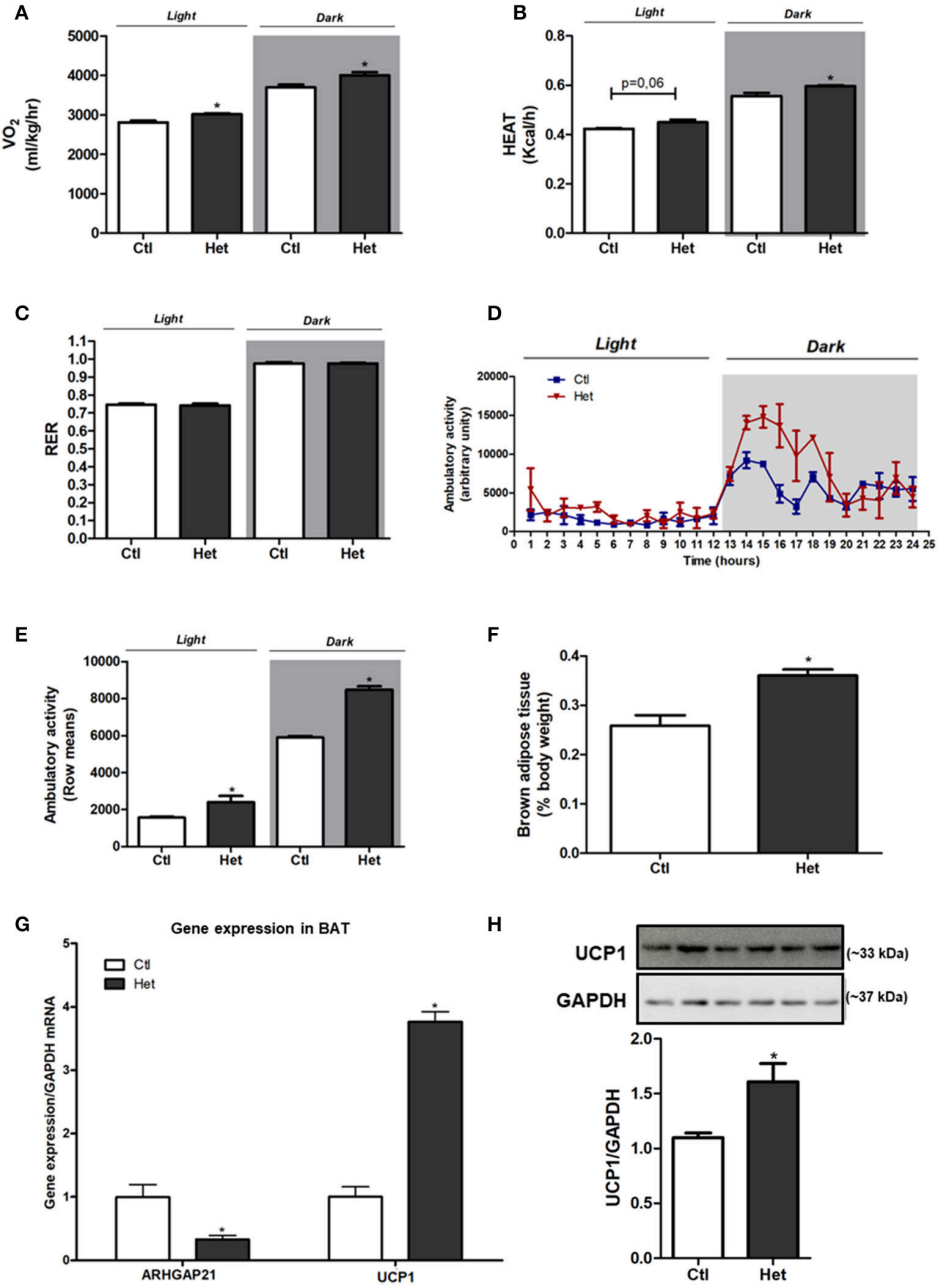
Energy homeostasis of ARHGAP21 Het mice. VO_2_ **(A)** *n* = 3, heat rate **(B)** *n* = 3 and respiratory exchange ratio (RER) **(C)** *n* = 3. Ambulatory activity for 24 h during light and dark periods **(D)** and mean of light and dark periods **(E)** *n* = 3. BAT weight **(F)** *n* = 6. Real-time PCR assay of ARHGAP21 and UCP1 mRNA levels in BAT **(G)** *n* = 3. Brown adipose tissue UCP1 protein content **(H)**, *n* = 3. The samples were transferred to nitrocellulose membranes in this sequence: Ctl, Het, Ctl, Het, Ctl, and Het. Control mice (Ctl) and ARHGAP21-haplodeficient mice (Het) fed a chow diet for 10 weeks. Data are the mean ± SEM. *P ≤ 0.05 (Student's-*t*-Test).

Het mice presented higher energy expenditure, as judged by the augmented VO_2_ (Figure 2A) and increased heat rate (Figure 2B) during dark and light periods, than did Ctl mice. No difference was found in RER between groups (Figure 2C). The ambulatory activity was significantly higher in Het than in the Ctl group in both periods (Figures 2D,E). BAT weight (Figure 2F), UCP1 mRNA expression (Figure 2G) and protein content (Figure 2H) were higher in Het mice than in Ctl mice. This accords with the higher energy expenditure observed in Het mice. A decrease in ARHGAP21 mRNA content in the BAT of Het mice was also observed (Figure 2G).

### Anorexigenic Effects of Whole-Body ARHGAP21 Reduction in Het-HFD Mice

**Figure 3:**
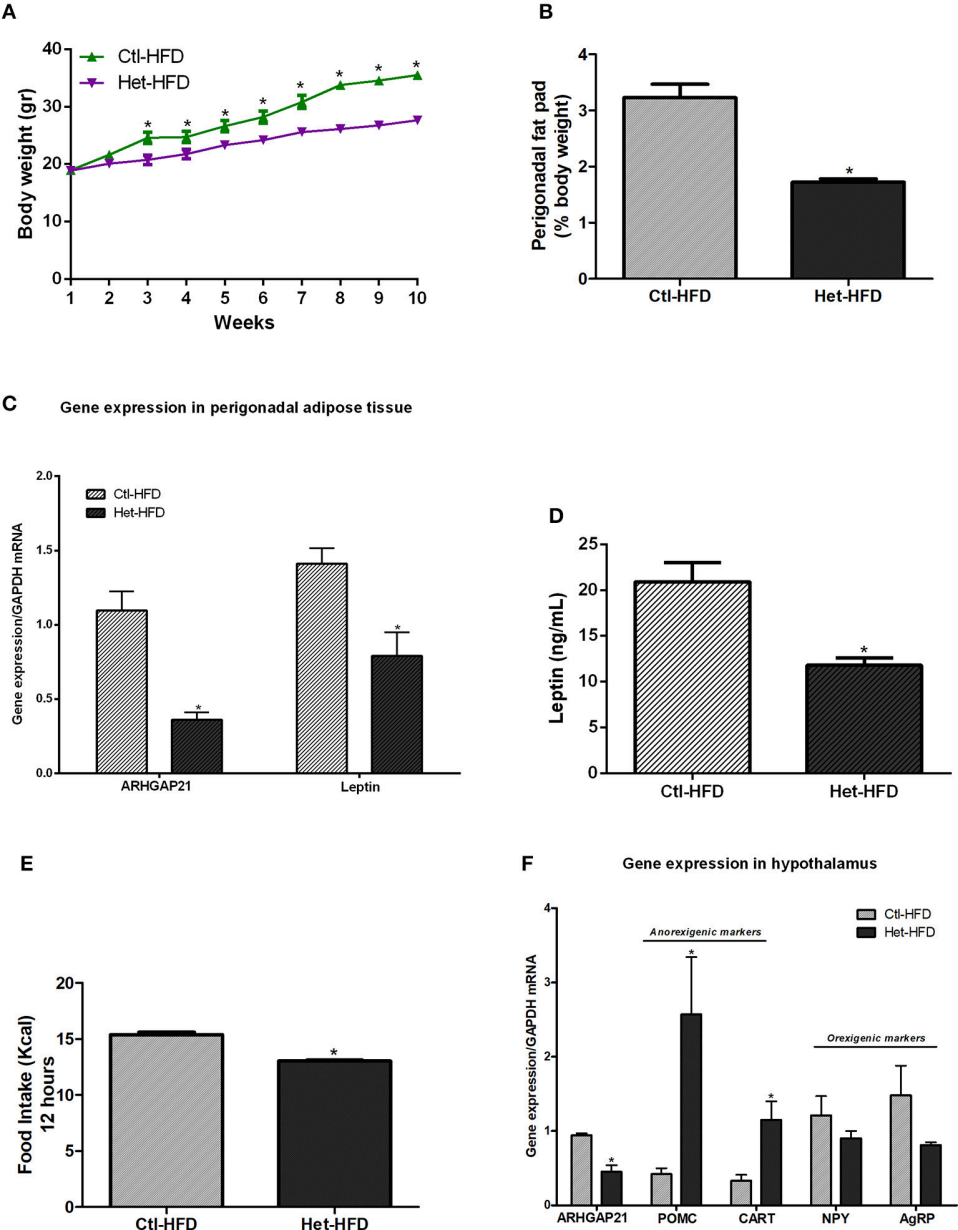
Anorexigenic effects of whole-body ARHGAP21 reduction in Het-HFD mice. Body weight curve **(A)** *n* = 6. Perigonadal fat pad weight **(B)** *n* = 6. Real-time PCR assay of ARHGAP21 and leptin mRNA levels in perigonadal adipose tissue **(C)** *n* = 6. Serum leptin level **(D)** *n* = 6. Food intake over 12 h **(E)** *n* = 3–5. Real-time PCR assay of hypothalamic ARHGAP21, POMC, CART, NPY, and AgRP mRNA levels **(F)** *n* = 5–6. Control mice (Ctl) and ARHGAP21-haplodeficient mice (Het) fed a high-fat diet for 10 weeks. Data are the mean ± SEM. *P ≤ 0.05 (Student's-*t*-Test).

We also challenged Het mice to a high-fat diet. As shown in Figure 3A, Het-HFD mice displayed lower body weight from the 3rd week until the end of the experimental period, accompanied by a decrease in perigonadal fat pad weight (Figure 3B), than did the Ctl-HFD group. In addition, leptin gene expression in the perigonadal adipose tissue (Figure 3C) and leptin serum levels (Figure 3D) were reduced in Het-HFD, compared with Ctl-HFD mice. We also found that the ARHGAP21 mRNA content was lower in the perigonadal adipose tissue of Het-HFD mice (Figure 3C). Het-HFD mice had less food intake (Figure 3E) and presented higher levels in the mRNA of anorexigenic markers (POMC and CART) than did Ctl-HFD mice (Figure 3F). However, NPY and AgRP mRNA levels were not different between groups (Figure 3F). Again, the expression of ARHGAP21 mRNA in the hypothalamus of Het-HFD mice was lower than in Ctl-HFD mice (Figure 3F).

### Energy Homeostasis of ARHGAP21 Het-HFD Mice

**Figure 4:**
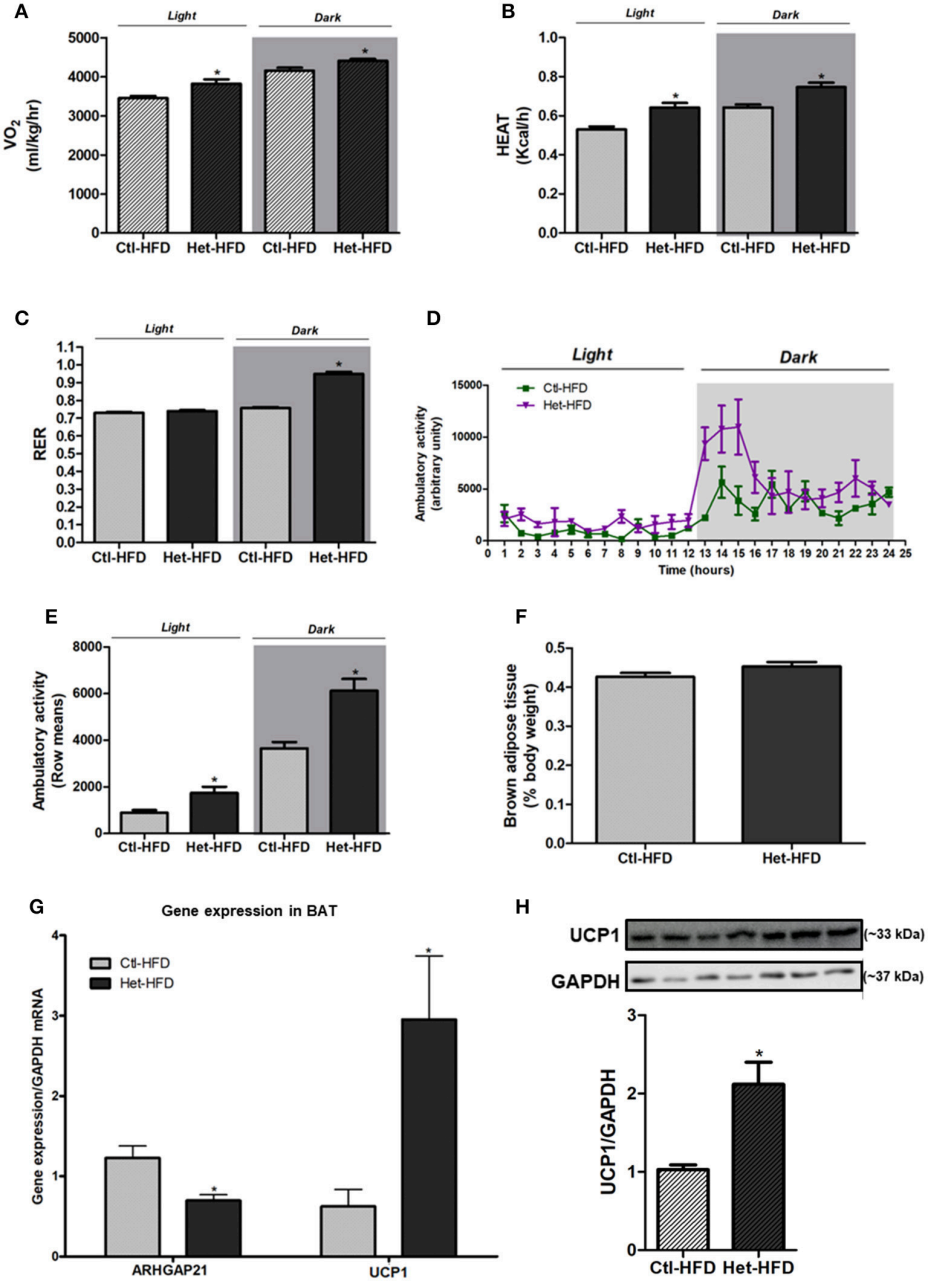
Energy homeostasis of ARHGAP21 Het-HFD mice. VO_2_ **(A)** *n* = 3, heat rate **(B)** *n* = 3, and RER **(C)** *n* = 3. Ambulatory activity for 24 h during light and dark periods **(D)** and mean of light and dark periods **(E)** *n* = 3. BAT weight **(F)** *n* = 6. Real-time PCR assay of ARHGAP21 and UCP1 mRNA levels in BAT **(G)** *n* = 4. Brown adipose tissue UCP1 protein content **(H)**, *n* = 3–4. The samples were transferred to nitrocellulose membranes in this sequence: Ctl-HFD, Het-HFD, Ctl-HFD, Het-HFD, Ctl-HFD, Het-HFD, and Het-HFD. Control mice (Ctl) and ARHGAP21-haplodeficient mice (Het) fed a high-fat diet for 10 weeks. Data are the mean ± SEM. *P ≤ 0.05 (Student's-*t*-Test).

The VO_2_ (Figure 4A) and heat rate (Figure 4B) were higher in Het-HFD than in Ctl-HFD during dark and light periods. Moreover, the RER of Het-HFD mice was higher during the dark phase, suggesting that they predominantly used carbohydrate oxidation in this period, as opposed to the Ctl-HFD mice that displayed metabolic inflexibility (Figure 4C). In addition, the ambulatory activity was significantly higher in the Het-HFD mice than in the Ctl-HFD mice (Figures 4D,E). BAT weight was similar between the groups (Figure 4F); however, we observed higher UCP1 mRNA expression (Figure 4G) and protein content (Figure 4H) in BAT of Het-HFD than in the Ctl-HFD group. Finally, Het-HFD mice had lower ARHGAP21 mRNA levels in the BAT than did the Ctl-HFD group (Figure 4G).

## Discussion

The inhibition of GAP TBC1D1 reduced the body weight and increased the energy expenditure in mice (16–20). Here, we extended these findings, showing a beneficial effect of whole-body reduction of ARHGAP21 in energy homeostasis, as judged by the increased energy expenditure and reduced food intake in both control and high-fat diet groups. These results were the first to point to possible involvement of a GAP family member in the control of food intake via increasing the expression of mRNA of the hypothalamic anorexigenic markers (POMC/CART). All these phenomena favor the reduction in body weight observed in ARHGAP21-haplodeficient mice.

We previously showed that ARHGAP21 inhibition decreased body weight in mice (21), but the mechanisms involved remain unknown. It is well established that body weight and appetite control are complex and that central mechanistic disturbances can lead to hyperphagia or anorexia depending on the balance between the expression of anorexic and orexic genes in the hypothalamus (7, 28). These genes are regulated by hormones, such as leptin and insulin, which increase the expression of anorexigenic genes in the hypothalamus, reducing food intake and increasing energetic expenditure (29–31). Exposition to high leptin levels, as observed in Ctl-HFD mice, contribute to leptin resistance through a negative feedback mechanism (32). Some practices and therapies as physical exercise and drug treatment can reduce leptin levels in obesity models, and this effect is associated with improvement in leptin signaling (33, 34). In this study, Het-HFD mice presented reduced adipose tissue pads and leptin levels, probably associated with improved leptin signaling in hypothalamus, in accordance with the metabolic improvement observed in this group. Moreover, we observed reduced food intake in Het and Het-HFD mice, corroborated by higher mRNA POMC and CART expression in the hypothalamus of these mice. In this context, ARHGAP21 inhibition improved energetic metabolism both by increasing anorexigenic gene expression in hypothalamus of Het and Het-HFD, and reducing fat pads and hyperleptinemia in Het-HFD.

POMC and CART peptides also stimulate energy expenditure (35). In fact, genetic ablation of POMC and CART in obese rodents was associated with reduced physical activity and energy expenditure (36). Conversely, intra-cerebroventricular (ICV) administration of CART in rodents induced an opposite effect (37). In agreement with these findings, we found that ARHGAP21-haplodeficient mice increased ambulatory activity and consequently energy expenditure, as judged by higher VO_2_ and heat rates than their respective controls. Despite reduced number of animals in the experiments of energy expenditure be a limitation of the study, the data from these experiments are in accordance with the molecular alterations observed in ARHGAP21 haplodeficient mice, such as, increase in hypothalamic anorexigenic genes and increase in UCP1 gene and protein expression in BAT, once these effects can be associated with increased energy expenditure.

The hypothalamic expression of POMC and CART is also known to increase energy expenditure independent on their effect on ambulatory activity. These peptides stimulate specific central neurons that, via the efferent sympathetic branch, stimulate thermogenesis by increasing mitochondrial uncoupling in adipose tissue (38–40). Accordingly, here, we observed increased UCP1 mRNA levels and protein content in the BAT of Het and Het-HFD mice. Our results reinforce the previously described involvement of a Rho-GAP family protein, DLC1, as a regulator of the adipocyte phenotype (41).

Moreover, we evaluated the RER, a measure widely utilized to evaluate the metabolic flexibility. The RER was calculated by measuring the amount of carbon dioxide (CO_2_) produced in comparison to the amount of oxygen (O_2_) used, and it is possible to predict which substrate is being oxidized as a fuel. During the light period, when mice are at rest and fasting, the RER is ~0.7, indicating a predominant use of fatty acid. On the other hand, during the dark period, when they are more active and fed, the RER is ~1.0, suggesting that they are using predominantly carbohydrate oxidation (42, 43). In some pathologies, such as, obesity and diabetes, the organism display metabolic inflexibility due to the incapacity to adjust uptake of macronutrients according to the metabolic needs (43, 44). In our study, as expected, mice submitted to high-fat diet presented metabolic inflexibility. However, Het-HFD mice presented increased RER during the dark phase, suggesting an improvement in metabolic flexibility, which can be explained, at least in part, by increased insulin sensitivity (21) leading to efficient glucose uptake and oxidation. These data support the hypothesis that ARHGAP21 reduction is able to boost the nutrient handling, energetic homeostasis, and metabolic flexibility (42, 43).

In summary, our study provides evidence supporting the beneficial effects of ARHGAP21 reduction upon energetic homeostasis, reducing food intake and increasing energy expenditure. Altogether, these events contributed to a reduction in body weight even in mice fed a high-fat diet. Thus, ARHGAP21 protein emerges as an important candidate to be considered for the prevention and treatment of obesity and associated diseases.

## Ethics Statement

All experiments involving animals were approved by the Animal Care Committee at UNICAMP (approval number: 3783-1).

## Author Contributions

GS and HB-S conceived and designed research. GS and LZ performed experiments. GS, LZ, JC-J, JV, and HB-S analyzed data. GS, LZ, JC-J, JV, and HB-S interpreted results of experiments. GS and HB-S prepared figures. EC and AB contributed to reagents, materials, and analysis tools. SS provided the knockdown animals used in the experiments. GS, JC-J, and JV drafted manuscript. GS, AB, and HB-S edited and revised manuscript. GS, LZ, JC-J, JV, EC, SS, AB, and HB-S approved final version of manuscript.

### Conflict of Interest Statement

The authors declare that the research was conducted in the absence of any commercial or financial relationships that could be construed as a potential conflict of interest.
